# Delayed-Onset Muscle Soreness Begins with a Transient Neural Switch

**DOI:** 10.3390/ijms26052319

**Published:** 2025-03-05

**Authors:** Balázs Sonkodi

**Affiliations:** 1 Department of Health Sciences and Sport Medicine, Hungarian University of Sports Science, 1123 Budapest, Hungary; bsonkodi@gmail.com; 2 Department of Sports Medicine, Semmelweis University, 1122 Budapest, Hungary

**Keywords:** delayed-onset muscle soreness, eccentric contraction, Piezo2, channelopathy, proton, quantum mechanical free energy stimulation, force production

## Abstract

Unaccustomed and/or strenuous eccentric contractions are known to cause delayed-onset muscle soreness. In spite of this fact, their exact cause and mechanism have been unknown for more than 120 years. The exploration of the diverse functionality of the Piezo2 ion channel, as the principal proprioceptive component, and its autonomously acquired channelopathy may bring light to this apparently simple but mysterious pain condition. Correspondingly, the neurocentric non-contact acute compression axonopathy theory of delayed-onset muscle soreness suggests two damage phases affecting two muscle compartments, including the intrafusal (within the muscle spindle) and the extrafusal (outside the muscle spindle) ones. The secondary damage phase in the extrafusal muscle space is relatively well explored. However, the suggested primary damage phase within the muscle spindle is far from being entirely known. The current manuscript describes how the proposed autonomously acquired Piezo2 channelopathy-induced primary damage could be the initiating transient neural switch in the unfolding of delayed-onset muscle soreness. This primary damage results in a transient proprioceptive neural switch and in a switch from quantum mechanical free energy-stimulated ultrafast proton-coupled signaling to rapid glutamate-based signaling along the muscle–brain axis. In addition, it induces a transient metabolic switch or, even more importantly, an energy generation switch in Type Ia proprioceptive terminals that eventually leads to a transient glutaminolysis deficit and mitochondrial deficiency, not to mention a force generation switch. In summary, the primary damage or switch is likely an inward unidirectional proton pathway reversal between Piezo2 and its auxiliary ligands, leading to acquired Piezo2 channelopathy.

## 1. Introduction

Delayed-onset muscle soreness (DOMS) is a delayed-onset pain sensation usually due to unaccustomed and/or strenuous eccentric or isometric muscle contractions. No single theory or cause of DOMS has been validated in more than a century. Correspondingly, several theories are running to explain the evolvement of this mysterious pain condition, like the lactic acid, muscle spasm, inflammation, connective tissue damage, muscle damage and enzyme efflux theories [1].

One recent neurocentric theory, the non-contact acute compression axonopathy theory of DOMS [2], encompasses all these aforementioned theories, but from a neural angle. Accordingly, DOMS may start off as a neural microdamage of the proprioceptive Type Ia afferent terminals within the muscle spindle [2]. One result of this suggested primary intrafusal painless microdamage is impaired proprioception, which could lead to a painful, harsher tissue damage in the extrafusal space when unaccustomed or strenuous eccentric contractions are excessively prolonged [2]. Not long after the description of this theory, the non-contact or autonomous microdamage of an ion channel, namely, Piezo2, was suggested to be the critical initiator of this proprioceptive microdamage in the primary damage phase of the suggested bi-phasic and bi-compartmental injury mechanism in DOMS [3]. Worthy to note that Piezo2 was shown to be the principal mechanosensory ion channel responsible for proprioception [4], and mechanotransduction is the conversion of external physical cues into internal biological and chemical ones. For years, scientists were skeptical about whether this protein could be microdamaged in an acquired fashion, as was proposed for autonomously acquired Piezo2 channellopathy in 2021 [3]. However, lately, more and more scientists are focusing on this hypothesis [5,6].

The above-mentioned microdamage of Piezo2 in primary proprioceptive afferent terminals within the muscle spindle could be analogous to the explosion of a protective “airbag” and resultant lost proprioceptive or injury protection. The impairment of this intrafusal (within the muscle spindle) proprioceptive protection may be followed by the aforementioned harsher secondary tissue damage within the extrafusal (outside the muscle spindle) space if the damaging eccentric contractions are sustained after the primary damage. It is noteworthy that this concept of a primary and a secondary damage was first introduced by Morgan et al. for extrafusal muscle fibers [7]. This bi-phasic concept was supported by an earlier observation of Fridén et al. that muscle fibers went through morphological abnormalities right after eccentric exercise, and these abnormalities were gradually extended to other muscle fibers in the next 2–3 days post-exercise [8]. However, Sonkodi et al. argued that the critical chain of the microdamaging events under prolonged forced lengthening or eccentric contractions starts at proprioceptive neuron terminals in the muscle spindle and not in muscle fibers in the extrafusal space [2].

Indeed, eccentric contractions involve higher cortical excitability with a lower motor unit discharge and higher force production than concentric contractions [9,10], and enhanced proprioceptive primary afferent loading is suggested when eccentric contractions are prolonged in order to support postural control [11]. In support of this, muscle fibers have a good aptitude to stretch, in contrast to neurons, which are far from being so resilient against stretch, compression and indentation. This difference in stretch resistance between the two tissues underlies the critical chain of events in this neurocentric microdamage process under forced lengthening contractions. Moreover, the theory of Sonkodi et al. entails both the intrafusal compartment as the locus of primary damage and the extrafusal compartment as the place of secondary damage. Hence, the neurocentric DOMS concept is not only bi-phasic, but bi-compartmental as well [2].

Weerakkody et al. demonstrated earlier that large fiber proprioceptive sensory neurons in the muscle spindle are involved in DOMS [12,13]. However, Mizumura and Taguchi found methodological errors in their research [14]. Later, Torres et al. also provided a demonstration of muscle spindle-derived reduced proprioception in DOMS, but rather attributed this eccentric exercise-induced microdamage to intrafusal muscle fibers and not to neurons [15]. As an important underlying mechanism of DOMS, eccentric exercise is known to damage proprioception [16]. In support of this, it was theorized that the static phase firing encoding of the stretch reflex is switched from the Piezo2-containing intrafusal Type Ia fibers to the acid-sensing ion channel 3 (ASIC3)-containing intrafusal Type II fibers due to the aforementioned intrafusal primary damage [11,17]. As a result of this switch, the medium-latency response (MLR) of the stretch reflex is delayed in DOMS, leading to impaired proprioception [11,17]. More recently, Espino et al. showed that miswired proprioceptive afferents indeed cause connection to inappropriate motoneuron pools; however, they demonstrated this invisible abnormality through the deletion of the Nav1.1 ion channel, a part of proprioceptive encoding in the primary afferents [18]. The authors of this manuscript suggest that an acquired channelopathy of the principal proprioceptive Piezo2 ion channel in the primary afferents could impair the function of Nav1.1, leading to miswiring or to a switch to the secondary proprioceptive afferents [18,19] and, hence, to connection to inappropriate motoneuron pools.

For this reason, the current paper aims to demonstrate why the initiating primary damage in DOMS or the acquired proprioceptive terminal Piezo2 microdamage could be viewed as a transient neural switch, leading to the unfolding of DOMS.

## 2. Proprioceptive Switch

Kouzaki et al. showed in their electromyography study that eccentric exercise-induced muscle damage increases the M-wave latency [20]. They considered this transient microdamage as extrafusal, hence arising from motoneurons and not from the muscle spindle [20].

It is noteworthy that Bennet et al. suggested a novel somatosensory terminal lesion, called terminal arbor degeneration (TAD), which could be observed as a result of paclitaxel chemotherapy [21]. Correspondingly, Vincent et al. demonstrated that platinum-analogue chemotherapy induced a complex intrafusal Type Ia proprioceptive impairment in either a transient or a chronic fashion [22,23]. It is noteworthy that a recent paper attributes the initiation of this proprioceptive impairment to a platinum-induced proton affinity switch in Piezo2 in proprioceptive Type Ia terminals [24]. Underpinning this proprioceptive impairment, Alvarez et al. and Bullinger et al. demonstrated the central synaptic disconnection of proprioceptors and the loss of vesicular glutamate transporter (VGLUT) 1/Ia synapses in motoneurons as a result of nerve injury [25,26]. Sonkodi et al. hinted that the intrafusal origin of increased M-wave latency in extrafusal motoneurons after eccentric exercise-induced muscle damage should not be ruled out [17]. They suggested that the acutely impaired intrafusal primary Type Ia proprioceptors and the consecutive VGLUT1 disconnection in motoneurons are the cause of the increased M-wave latency in DOMS [17]. Furthermore, Sonkodi et al. also demonstrated that, as a result of this microdamage, the MLR of the stretch reflex was indeed significantly delayed in DOMS [17]. Later, McIntosh et al. showed in the SOD1-G93A mouse model of amyotrophic lateral sclerosis (ALS), as the delay in the MLR in DOMS was first proposed through an ALS-based theory [11], that abnormalities first evolve in neuromuscular junctions in the form of dysfunction, but later even postsynaptic structures detach from the neuromuscular junctions, and all happens before the appearance of ALS motor symptoms [27].

**Figure 1:**
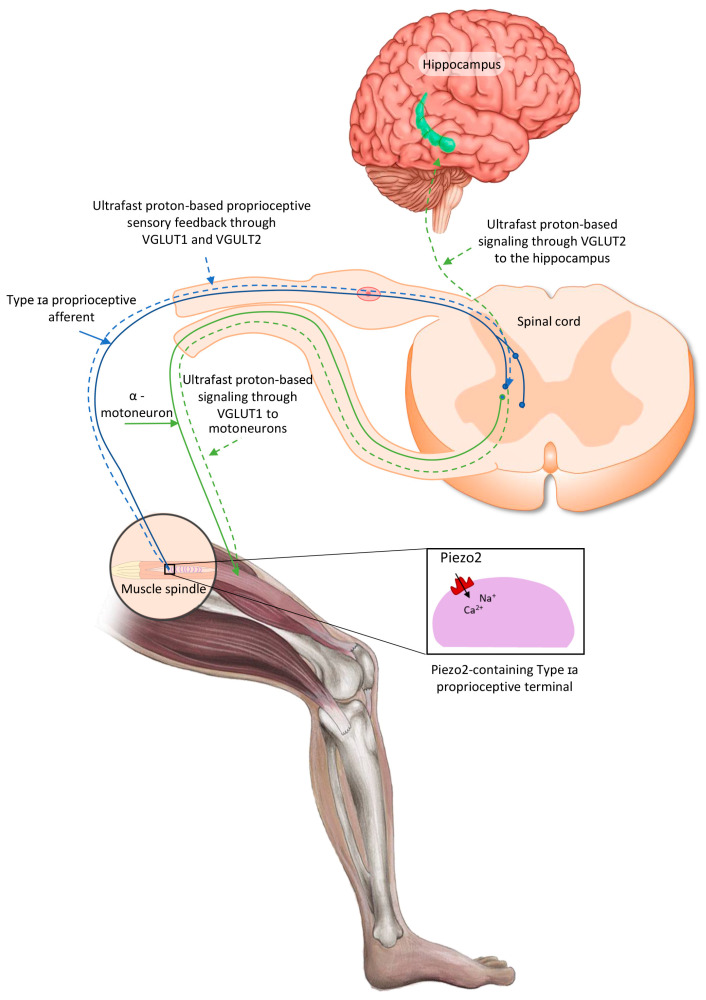
Proposed intrafusal proprioceptive terminal Piezo2-initiated ultrafast proton-based long-range synchronization to hippocampal theta rhythm through VGLUT2—the current figure is an English adaption of the figure from *Hungarian Rheumatology* [37].

Recently, it has been theorized that the activation of the intrafusal Piezo2 ion channel in the Type Ia terminals not only could initiate an ultrafast proton-signaled proprioceptive feedback to motoneurons, but also could initiate an ultrafast proton-signaled long-range synchronization to the hippocampal theta rhythm through VGLUT2 (Figure 1), constructing an ultrafast muscle–brain axis [28,29,30,31]. It is noteworthy that this theory could be viewed as a more definite extension of the earlier coupled oscillator model of Cathers et al., who suggested the entrainment of the imposed forcing intrafusal Type Ia afferent peripheral oscillator to the oscillator(s) of the central nervous system [32]. Later, it was also proposed that the acquired channelopathy of Piezo2 in Type Ia terminals could not only disconnect VGLUT1 in motoneurons, but also impair allosteric transmission regulation at a distance through VGLUT2 towards the hippocampal theta rhythm, in a transient fashion in DOMS and in a progressive and irreversible fashion in ALS [29]. It is important to highlight again that unaccustomed and/or strenuous eccentric contractions could induce DOMS. Therefore, this unaccustomed and/or strenuous exercise-induced VGLUT2 disconnection, as the consequence of the primary damage in DOMS, may impair the ultrafast spatial and even speed input to the hippocampal theta rhythm [29,31]. In support of this theory, the hippocampus is responsible for initial learning and spatial memory task retention [33,34,35]. It is noteworthy that a recent paper indicated the so-called swap error, i.e., the swapping of the location of recognized items, as a reflection of impaired short-term hippocampal memory [34,36]. This phenomenon is likely due to the proposed impairment of the ultrafast proton signaling in the muscle–brain axis due to the Piezo2 channelopathy [24].

One indirect proof of the proprioceptive Piezo2 channelopathy-derived lost ultrafast long-range proton-signaled synchronization towards the hippocampal theta rhythm, or the impairment of ultrafast signaling in the muscle–brain axis, in DOMS was provided by Keriven et al. They demonstrated that paired associative transcranial and peripheral electromagnetic stimulation could mitigate the symptoms of DOMS [28,38]. Additional substantiation of this theory was provided by the observation that solo peripheral electromagnetic stimulation, without transcranial stimulation, was ineffective in DOMS [39].

In summary, the sequence of events resulting in primary microdamage in DOMS is as follows. Allostatic stress-induced overexertion of the Type Ia proprioceptive terminal under repetitive forced lengthening contractions could lead to an autonomously acquired Piezo2 channelopathy [3,40]. This primary damage entirely switches the static-phase firing encoding to Type II intrafusal fibers, which is reflected in the delayed MLR of the stretch reflex [17]. Moreover, this primary damage, in association with impaired vesicular glutamate release, leads to impaired VGLUT1 connection in motoneurons and impaired VGLUT2 connection towards the synchronization with the hippocampal theta rhythm. Eventually, these proprioceptive switch/miswiring and impaired VGLUT1/2 connections will lead to the transiently increased M-wave latency in motoneurons and transiently dysfunctional neuromuscular junctions.

It is important to note that eccentric exercise-induced DOMS is generally considered to cause muscle microdamage, but Yu et al. showed with immunoelectron microscopy that these myofibrillar and cytoskeletal alterations rather represent the adaptive remodeling of myofibrils [41]. Accordingly, the current author suggests that transient dysfunctional neuromuscular junctions are to blame for this adaptive remodeling of myofibrils present in the ultrastructural enigmatic picture of DOMS, namely, Z-disc streaming, Z-disc smearing and Z-disc disruption, as indicated by Yu et al. [41]. It is notable that Sonkodi also viewed acquired Piezo2 channelopathy as the breach of remodeling [29,42] and the gateway from physiology to pathophysiology [3]. In support of this theory, Mizumura and Taguchi found no injured muscle fibers using light microscopy in their DOMS model and showed that damage could be provoked only in very high exercise settings [14]. It is remarkable that this finding is also in line with the neurocentric DOMS theory and may support the notion that nerves are less capable of stretching than muscles and, hence, more prone to microdamage.

Furthermore, the current author also proposes that the transient Piezo2 channelopathy-induced miswired motoneural or dysfunctional neuromuscular junctions may contribute to nerve growth factor (NGF) production by muscle cells and/or satellite cells and glial cell line-derived neurotrophic factor (GDNF) production in muscle cells. A significant finding is that NGF and GDNF are indeed involved in DOMS, as was first demonstrated by Mizumura and Taguchi [14]. However, this NGF and GDNF production could be the result of proprioceptive terminal Piezo2 channelopathy-derived switched signaling and impaired cross-frequency coupling of Piezo2–Piezo2 and Piezo2–Piezo1 [29,31], leading to impaired Piezo1-driven cell orientation and adjustment, as was suggested by Sonkodi et al. [42]. Accordingly, NGF and GDNF may induce the involvement of Type III and IV fibers in the extrafusal space as a backup or fortification of proprioception by ASIC3, which is present in these fibers [14]. ASIC3 could be considered as the ion channel responsible for compensatory or secondary proprioception [28,43,44] following primary proprioceptive Piezo2 microdamage [3,28,40] or the aforementioned explosion of the protective “airbag”. As a research-based verification, Khataei and Benson demonstrated the compensatory and protective proprioceptive role of ASIC3 in DOMS [45]. Further support derives from recent research that implicates Piezo2 as a negative regulator of neurotrophic factor release and shows that its deficiency is associated with elevated neurotrophic release [46]. This function of Piezo2 or, more importantly, its deficiency may explain why NGF and GDNF production is heightened in DOMS as a nerve microinjury-derived trophic signaling activation, most likely due to the aforementioned acquired Piezo2 channelopathy-induced impaired Piezo2–Piezo2 and Piezo2–Piezo1 crosstalk.

Increased blood flow-induced shear stress and arterial pressure pulsation are important factors in the activation of Piezo2 when acute intensive sport activities are initiated [47]. Recent research highly supports that Piezo2 might be capable of inducing an intrinsic oscillatory interoceptive mechanism through a pressure pulsation transduction pathway [48]. This Piezo2-derived interoceptive signaling modulates olfactory bulb activity in arousal, not to mention that it is synchronized to brain activities [48]. It is noteworthy that an analogous Piezo2-based synchronization mechanism was theorized earlier by Sonkodi [31,47]. Blood flow restriction is known to induce muscle damage and the repeated bout effect (RBE) in reference to DOMS [49]. Correspondingly, the current author proposes that muscle damage could be the result of blood flow restriction-derived lost pressure-pulse detection and lost Piezo2-initiated oscillatory synchronization. Hence, hypoxia-associated mechanoenergetic primary proprioceptive impairment or Piezo2 channelopathy-induced impaired oscillatory synchronization will prevail and also lead to the secondary damage phase of DOMS and RBE if reinjury is introduced again.

## 3. Ultrafast Signaling and Transcription Switch

Auxiliary protein subunits of Piezo2 may also have a role in the development of its channelopathy, since allostatic stress could induce the aforementioned conformational changes in protein–protein interactions as well. One example is the myoblast determination protein 1 (MyoD)-family inhibitor protein [50]. The transmembrane protein 120A (TMEM120A), also called TACAN, is another auxiliary protein that inhibits Piezo2 [51]. Interestingly, TMEM120A contributes to leakage currents [52], and part of the Piezo2 channelopathy theory suggests that Piezo2 microdamage induces subthreshold leakage currents [3].

Activated N-methyl-D-aspartate (NMDA) receptors, following the aforementioned proprioceptive switch, were proposed earlier as the gate controllers of DOMS [11] in line with the gate control theory of pain [53]. In addition, this pain theory has long implicated wide-dynamic-range neurons (WDR neurons) in the spinal dorsal horn [54,55]. Accordingly, WDR neurons could be activated by NMDA receptors in conjunction with L-type calcium currents and calcium-activated nonspecific cationic currents [56]. Proprioceptive acquired Piezo2 channelopathy, as the primary damage in DOMS, is proposed to induce NMDA receptor activation due to the associated impairment in the vesicular glutamate release machinery [3,40]. Moreover, it also induces L-type calcium currents as a direct result of the transiently lost functionality of Piezo2 and calcium-activated nonspecific cationic currents due to the suggested dissociation of TMEM120A, leading to imbalanced subthreshold leakage currents [19]. In support of this theory, Piezo indeed contributes to L-type calcium current negative modulation [57].

The aforementioned WDR neurons participate in the pain sensitization mechanism in the spinal dorsal horn [58]. In support of this observation, loss-of-function mutations in PIEZO2 lead to loss of pain and sensitization [59]. Indeed, the primary damage phase or Piezo2 channelopathy in the absence of the secondary damage phase in DOMS is suggested to be a pain-free condition [3]. It is important to note that ASIC3 also has the capability to activate the WDR neurons in the spinal dorsal horn [60]. Therefore, activated ASIC3 in intrafusal Type II fibers and, later, in Type III fibers, could also contribute to the initial activation of the WDR neurons due to the autonomously acquired Piezo2 channelopathy-induced proprioceptive switch [19].

It is noteworthy that the suggested impairment in the ultrafast Piezo2-initiated proton-signaled long-range pathway in the muscle–brain axis to the hippocampus will result in another switch, namely, from proton-coupled signaling through VGLUT2 to glutamate-based signaling [31]. This switch from proton to glutamate signaling may even result in the crosstalk with nociceptive C-fibers if the secondary damage phase is present [2,3,29]. However, this signaling will not be ultrafast anymore, though still rapid. In support of this, the hippocampus is indeed one of the central regions where pain signals are modulated [61].

Neural stem cells sense excitatory neural activity through L-type Ca^2+^ channels and activated NMDA receptors, leading to changes in gene expression and neurogenesis [62]. Osteocalcin had also been suggested to have a role in this process [31], as the non-contact acute compression axonopathy theory of DOMS proposes that osteocalcin could be responsible for inducing the acute stress response involved in the primary damage phase of DOMS [2]. In support of this, physical exercise is indeed capable of increasing the activation of immediate early genes in the hippocampus, and no other part of the brain can do so [63]. Furthermore, the hippocampus is a key region for arousal, stress regulation and anxiety [63], not to mention that there is a direct link between stress signaling and hippocampal theta rhythm [64].

Mizumura and Taguchi rightly introduced their earlier and recent significant findings in their paper in reference to NGF and GDNF [14]; however, these factors most likely play a role only in the secondary damage phase of DOMS, following the primary damage [44]. Accordingly, cyclooxygenase-2 (COX2) upregulation of GDNF is an essential process in DOMS [65]. It is noteworthy that syndecan-3 is a novel receptor for GDNF [66]. Therefore, syndecan-3 signaling could be mitigated by GDNF in Type III sensory terminals under sensory hyperexcitation. In addition, GDNF and NGF also crosstalk extrafusally [67]. Moreover, NGF sensitizes nociceptive C-fibers in DOMS through COX2 and bradykinin [68], hence leading the way to pain sensation and neuroinflammation in a critical way [69]. Interestingly, Ota et al. demonstrated that the transient receptor potential 1 (TRPV1) ion channel in nociceptive Type IV neurons also contributes to DOMS in the extrafusal muscle space [70] and that TRPV1 could also induce the WDR neuron response as a result of tissue injury [71]. After all, the crosstalk and cross-activation of all four types of overexcited muscle-derived somatosensory neurons, namely, Type Ia, Type II, Type III and Type C fibers, are essentially involved in the unfolding of DOMS [2,40]. Moreover, the crosstalk and cross-activation initiator is suggested to be the Piezo2 channel [31,42].

Finally, Piezo2 microdamage is suggested to be accompanied by the aforementioned imbalanced subthreshold leakage currents [3]. Indeed, intracellular calcium currents are more sensitive to protons than extracellular ones [72]. Therefore, it is likely that the conformational change in TMEM120A, leading to its functional dissociation from Piezo2 under allostatic stress and the resultant undesired proton leak into the intracellular space, may explain the presence of intracellular imbalanced subthreshold leakage calcium currents when they should not be present [29,30,31]. It has been suggested that these imbalanced subthreshold leakage calcium currents may be the calcium-activated nonspecific cationic currents that participate in WDR neuron activation in the spinal dorsal horn [29]. Further in support of this theory, TMEM120A contributes to mechanical hyperalgesia [73], and it is worthy to note that DOMS is a mechanism of muscular mechanical hyperalgesia. Moreover, it is also likely that the conformational change in the MyoD-family inhibitor protein, leading to its functional dissociation from Piezo2 under allostatic stress and the resultant undesired proton leak into the intracellular space, may explain transcription activation. This is in line with the proposition that Piezo2 channelopathy is a principal transcription activator [19].

## 4. Metabolic and Energy Generation Switch

The autonomously acquired Piezo2 channelopathy was also suggested to cause impaired cross-frequency coupling between proprioceptive Piezo2 and Piezo1 in peripheral cells through lost Huygens synchronization in a given compartmental micromilieu involving mitochondria and protons [31,42]. Furthermore, this proprioceptive ion channel microdamage was also proposed to impair not only Piezo2–Piezo2 crosstalk between the intrafusal and the extrafusal space, but also Piezo2–Piezo2 crosstalk with the autonomic nervous system [47]. As a result, transient dysautonomia may prevail [3]. In support of this theory, Sonkodi et al. demonstrated in a pilot study with an orthostatic stress test in competitive swimmers that orthostatic tolerance was reduced after DOMS-inducing exercise [74]. Moreover, this orthostatic impairment resembled the one experienced in diabetic patients [74], involving abnormal diastolic blood pressure and heart rate [75]. Further in line with these findings, it was observed that the greater was the eccentric exercise-induced muscle damage reflected in the plasma level of creatine kinase, the greater was insulin resistance [76]. Sonkodi et al. attributed this negative correlation to the action of the atypical hippocampal-like metabotropic glutamate receptor (mGluR) phospholipase D (PLD) complex in primary afferents, identified by Thomson et al. [77], which is a homomeric excitatory ionotropic glutamate receptor (GluK2) [77]. In fact, Gluk2 has a role in the regulation of glucose homeostasis [78]. However, this negative homeostatic regulation could be derailed by the acquired Piezo2 channelopathy-induced DOMS effect. As a result, impaired Piezo2–Piezo2 crosstalk-induced autonomic dysregulation and impaired Piezo2–Piezo1 crosstalk could prevail, resulting in insulin resistance, because Piezo1 has a role in glucose-induced insulin secretion [79]. Another consideration in addition to this negative homeostatic glucose regulation is that eccentric contractions may induce lactate consumption by proprioceptive afferent terminals in the muscle spindle through a proposed astrocyte–neuron lactate shuttle (ANLS)-like mechanism [43]. Furthermore, proprioceptive terminal Piezo2 channelopathy, due to fatigue-inducing eccentric contractions under allostatic stress, is suggested to be associated with impairment in vesicular glutamate release [17], which could be the direct result of the impairment in the aforementioned ANLS-like machinery [43]. Hence, Piezo2 channelopathy is not only a proprioceptive switch or miswiring mechanism, but also a metabolic switch, as was indicated by Sonkodi, in DOMS and ALS [29].

Another metabolic alteration due to eccentric exercise-induced muscle damage regards lipid markers, as the higher the magnitude of the muscle damage, the lower the levels of lipid markers [76]. One explanation for this phenomenon could be that the activation and excitation of Piezo ion channels could lead to lipid and cholesterol depletion [40]. This depletion could be likely due to the fact, recently observed, that Piezo pore opening creates interhelical gaps that are abruptly filled with lipids in order to sustain pore conduction [80]. Moreover, Maughan et al. showed increased lipid peroxidation right after DOMS-inducing downhill running, a typical eccentric exercise, which later increased further and peaked 6 h after the exercise [81]. The emblematic propeller blade in Piezo2 structure is in fact neighbored by negatively charged membrane lipids [82,83,84], and protein–lipid interaction could endure conformational alterations under allostatic stress. Correspondingly, phosphatidylinositol 4,5-bisphosphate (PIP2) depletion could activate phospholipase C (PLC), and the resultant activation of TRPV1 could inhibit Piezo ion channels [85]. Moreover, PLD produces phosphatidic acid, which could also inhibit Piezo2 [86]. Mechanical shear stress could cause disruption in the nanodomains of lipid rafts with a resultant interaction between PLD and activated PIP2 [87]. It is noteworthy that PLD and phosphatidic acid might have a role in the biogenesis and cargo loading of extracellular vesicles as well [88]. This could be relevant in light of the Piezo2 channelopathy theory, because it is suggested to be associated with impairment of vesicular glutamate release [3,19].

In summary, these protein–lipid conformational changes are worthy of note, since it seems that PLD might have role in Piezo2 inhibition of Type Ia proprioceptive terminals within the muscle spindle. In support of this, a recent paper suggested the role of Piezo2 and proprioceptive primary afferent terminals containing atypical hippocampal-like mGluR coupled to PLD in the Piezo2-initiated proton-signaled long-range ultrafast synchronization to the hippocampal theta rhythm [29]. On the contrary, PLC may participate in the inhibition of Piezo1 and Piezo2 in the extrafusal muscle compartment.

This transmembrane and extracellular membrane surface lipid depletion phenomenon was emphasized in DOMS by Sonkodi, who suggested that this is why the dual COX2 and lipoxygenase inhibitory function of diclofenac is effective in the prevention of DOMS evolvement [40]. Even more importantly, lipoxygenase could be considered as a catalyst of the proton-coupled electron transfer (PCET) reaction and hydrogen tunnelling, besides catalyzing the deoxygenation of polyunsaturated fatty acids, including linoleic acid [89]. This effect is dependent on the local electrostatic micromilieu [89]. This local electrostatic field may promote conformational changes in the enzyme, and the resultant shorter donor–acceptor distance would be preferential for the aforementioned efficient hydrogen tunnelling and proton transfer [89]. The hydrogen atom abstraction capability of lipoxygenase would also facilitate the concerted proton tunneling–electron tunneling (PTET) reaction [90]. These tunnelling reactions are highly in support of the quantum mechanical/molecular mechanical free energy stimulation aspect of Piezo2-initiated ultrafast long-range proton signaling theory [29,31]. This is especially true when we consider the distance dependence due to the forced lengthening nature of DOMS-inducing eccentric contractions, or the stretch and poking/indentation detection capability of Piezo2 channels. Moreover, it is suggested that Piezo2 channelopathy is associated with impairment of the vesicular glutamate release [3] and the ANLS like machinery [43]. Interestingly, compartmentalized glutamate leakage activates the lipoxygenase pathway, leading to reversed glutamate transport, and promotes oxidative astrocyte death [91]. This is also in support of the acquired Piezo2 channelopathy theory [29,31]. Accordingly, the suggested transmembrane proton affinity switch or proton pathway reversal between Piezo2 and its auxiliary subunits will not only induce impairment of the vesicular glutamate release, but VGLUT1 disconnection as well [29,31].

Another interesting observation provided by Ochi et al. is that eicosapentaenoic acid-rich fish oil supplementation 8 weeks prior to DOMS-inducing eccentric exercise showed to be protective of motoneuronal function [92]. This dietary supplementation strategy significantly shortened M-wave latency, as well as mitigated the loss of force and reduced the range of motion and the pain intensity in DOMS [92]. This is suggestive of the role of lipids in reference to the neurocentric Piezo2 channelopathy theory according to which linolenic acid supplementation in Angelman syndrome alleviates the dysfunction [93] or the acquired channelopathy of Piezo2. Another interesting finding is that Piezo2 causes a voltage block in cell membranes and the Piezo2 channel itself is strongly modulated by membrane voltage [94]. Therefore, the negatively charged membrane lipid and protein conformational changes around Piezo2 may have high relevance under allostatic stress, leading to impaired voltage block function of Piezo2.

TMEM120A also has a role in lipid metabolism and in the regulation of the innate immune response [51]. Hence, TMEM120A may dissociate functionally from Piezo2 under allostatic stress when their interaction undergoes conformational changes in DOMS, leading to the proposed inducement of imbalanced subthreshold leakage currents [3,17], dysregulation of lipids [3,95] and dysregulation of the innate immune system [3], as was demonstrated in DOMS [96].

DOMS-inducing full-body resistance training was demonstrated to elevate resting energy expenditure for up to 72 h not only in untrained, but also in resistance-trained individuals [97]. The current author suggests that the underlying critical energy generation switch of the impairment of the ANLS-like mechanism in Type Ia proprioceptive terminals in DOMS [43] is due to the derailment of the mitochondrial oxidative phosphorylation system (OXPHOS). As a result of energy generation from oxidative phosphorylation, electrons are transferred within the mitochondrial electron transport system, and protons are translocated to the outer surface of the cell membrane. This process leads to chemical energy generation through adenosine triphosphate (ATP) production from the transfer of electrons and the proton motive force. Furthermore, it also explains the aforementioned quantum mechanical free energy stimulation.

However, when the aforementioned auxiliary subunit TMEM120A and/or MyoD go through conformational change and functional dissociation from Piezo2 in proprioceptive terminals under allostatic stress, as proposed in DOMS, inward leakage of extracellular protons or proton pathway reversal may occur. Hence, this microdamage not only impairs the theorized low-frequency Schottky barrier semiconductor diode-like feature of Piezo2 function [31], but also could impair the intracellular electrochemical proton gradient and efficient quantum mechanical free energy stimulation. Protons are not needed for glutamine uptake by neurons during the glutamine–glutamate cycle, since a low pH is enough [98]. However, the aforementioned impaired intracellular proton gradient could switch mitochondrial energy metabolism from the evolutionarily superior energy-generating OXPHOS and glutamine respiration pathways to the mitochondrial glucose and, even more importantly, glutamine fermentation pathways [24]. During fast growth, this glucose and glutamine respirofermentation may run simultaneously [99], like in. cancer and immune cells [99]. Indeed, Sonkodi et al. suspected that this special metabolic programming could be involved in the dysregulated increase in innate immune natural killer T cells (NKT cell) in DOMS [96]. Not to mention that Sonkodi et al. also suggested that “DOMS could play an important role in ontogenesis by triggering muscle growth and adapting the nervous system in the growth process” [2]. Accordingly, coaches often use DOMS as a muscle-gain trigger mechanism during training sessions under the credo of “no pain, no gain”.

After all, the impairment of the aforementioned ANLS-like mechanism in Piezo2-containing Type Ia proprioceptive terminals in DOMS [16] could be explained by this simultaneous switch to glucose and, more importantly, glutamine fermentation induced by the deficit in mitochondrial OXPHOS-dependent energy generation and glutaminolysis. It is noteworthy that glycolysis is essentially the preferred and more efficient energy (in the form of ATP) generation method, and respiration generates a higher ATP yield than fermentation [99]. This proposed energy-generating switch due to a glutaminolysis deficit is transient and lasts for up to 72 h or until the intracellular impaired proton gradient ceases due to the functional restitution of TMEM120A and/or MyoD as the auxiliary ligands of Piezo2. In support of this energy metabolism derailment is the fact that the expression of skeletal muscle mitochondria-related genes is affected in DOMS [100]. However, the current author proposes that this mitochondrial damage is initiated in the intrafusal proprioceptive terminals in the primary damage phase of DOMS due to the above-mentioned energy metabolism switch. Paradoxically, this primary damage-derived energy generation switch induces higher energy metabolism in the nervous and muscle system, because the proprioceptive switch involves increased neuron and muscle cell activation in circuitries as a compensatory mechanism. On the side of the above energy-generating switch it is long known that glutamine supplementation indeed attenuates strength loss and muscle soreness after eccentric exercise [101] and even mitigates muscle damage [102].

Nonetheless, genetic predisposition or environmental exposure, not to mention aging, may present an impediment to the full functional restitution of Piezo2 in proprioceptive terminals in ALS [19]. As a result of progressive dysfunctional mitochondria, energy generation is reduced in affected neurons and muscle cells in ALS [19]. Correspondingly, energy metabolism in muscles shows severe degradation in ALS with mitochondrial dysfunction [103]. However, it is proposed that the underlying pathomechanism is again initiated in the proprioceptive terminals of the muscle spindle [11] due to an irreversible autonomously acquired Piezo2 channelopathy [29,31,95]. In support of this, common- and rare-variant association analyses highlighted a cell-autonomous disease initiation in glutamatergic neurons in ALS [104], and it has been suggested earlier that intrafusal proprioceptive Type Ia fibers are these glutamatergic neurons [11] damaged by the progressive irreversible Piezo2 channelopathy [19,29,95,105]. Further substantiation of this energy generation switch-induced depletion and metabolic switch in ALS is provided by the lower level of ATP, higher level of mitochondrial uncoupling proteins, and higher lipid and carbohydrate metabolism in ALS [29,103].

## 5. Force Generation Switch

An unanswered question of science regards the mechanism of higher force generation and lower motoneuron excitability associated with eccentric contractions. It is noteworthy that eccentric contractions induce DOMS. The author of this manuscript posits that the above-proposed quantum mechanical free energy stimulation pathway could explain not only the above-mentioned high energy generation, but also the enigmatic high force generation capability of eccentric contraction.

It is indicative that a recent study found that a lower voluntary activation of eccentric contractions is partly due to lower corticospinal excitability [10]. Ruas et al. devoted the shorter corticospinal silent periods during eccentric contractions to extra muscle spindle-derived excitation and not to lower intracortical inhibition [10]. Accordingly, the suggested proton-coupled ultrafast quantum mechanical free energy stimulation provides the additional excitatory modulation directly to motoneurons through VGLUT1 and to the hippocampus through VGLUT2. Moreover, this may explain why motoneuron excitability is lower but force generation is higher in eccentric contractions, as opposed to isometric or concentric contractions.

In conclusion, the suggested acquired inward unidirectional proton pathway between Piezo2 and its auxiliary ligands, leading to Piezo2 channelopathy, impairs force production, which is one key symptom of DOMS.

## 6. Conclusions

The root cause of ageing or the “primary damage” is a long-searched research subject, but remains unexplained [106]. Recently, it was proposed that autonomously acquired Piezo2 channelopathy in proprioceptive nerve terminals could be the searched primary damage [3,29,31]. Even though it sounds esoteric, mechanotransduction, namely, the conversion of external physical inputs to internal biological and chemical ones, is miraculous, especially at an ultrafast pace, involving quantum mechanical free energy stimulation. Not to mention that it shifts the scientific focus to the protonic world of regulation, since scientists know that not all neurotransmission could be explained during acute intensive exercise activities by the action of known classic neurotransmitters. Intact ultrafast neurotransmission seems to be essential for competitive athletes under strenuous allostatic stress, not to mention under unaccustomed circumstances, to avoid DOMS.

Consequentially, other conditions and diseases are suspected to start off with the aforementioned primary damage or the acquired Piezo2 channelopathy in proprioceptive terminals, as suggested for DOMS. DOMS could be an excellent research model for neuroscientist to explore this pain-free initiating primary damage phase of a bi-phasic mechanism, because we have knowledge of how to induce DOMS. After all, it is not surprising that abusing DOMS and the RBE could lead to a chronic condition if DOMS-inducing eccentric contractions are exerted every day without an adequate regeneration periodization [107]. Therefore, in chronic conditions, we see only a chronic compensatory and depletory clinical picture with increased necrotic and regenerating cells, and it is hard to understand how the pathophysiology initiated.

However, our diagnostic tools are still too limited to investigate this proposed pain-free initiating primary damage phase, though neuroscience is accumulating findings in a fast pace to enhance our understanding in order to find new investigative angles and manipulate the proposed “switches”. One suggested consequence of DOMS is the transient switch from proton-coupled ultrafast signaling to rapid glutamate-based signaling along the peripheral muscle–brain axis. This switch is an especially intriguing area of future research, because the switch on this central link to the hippocampus, the hub for neurogenesis, could be one key mechanism leading to neurodegeneration or accelerated aging.

## Data Availability

Not applicable.

## Abbreviations

ALS	Amyotrophic lateral sclerosis
ANLS	Astrocyte–neuron lactate shuttle
ASIC3	Acid-sensing ion channel 3
ATP	Adenosine triphosphate
COX-2	Cyclooxygenase-2
GluK2	Ionotropic glutamate receptor
DOMS	Delayed-onset muscle soreness
GDNF	Glial cell line-derived neurotrophic factor
mGluR	Metabotropic glutamate receptor
MLR	Medium-latency response
MyoD	Myoblast determination protein 1
NGF	Nerve growth factor
NKT cells	Natural killer T cell
NMDA	N-methyl-D-aspartate
OXPHOS	Oxidative phosphorylation system
PCET	Proton-coupled electron transfer
PIP2	Phosphatidylinositol 4,5-bisphosphate
PLC	Phospholipase C
PLD	Phospholipase D
PTET	Proton-tunneling–electron-tunneling
RBE	Repeated bout effect
TAD	Terminal arbor degeneration
TMEM120A	Transmembrane protein 120A
TRPV1	Transient receptor potential 1
VGLUT	Vesicular glutamate transporter
WDR neurons	Wide-dynamic-range neurons
